# *In vitro*-engineered non-antibody protein therapeutics

**DOI:** 10.1007/s13238-017-0386-6

**Published:** 2017-03-07

**Authors:** Rudo Simeon, Zhilei Chen

**Affiliations:** Department of Microbial Pathogenesis and Immunology, Texas A&M University Health Science Center, College Station, TX 77845 USA

**Keywords:** scaffold, multivalent, phage, yeast, ribosome, antibody surrogate

## Abstract

Antibodies have proved to be a valuable mode of therapy for numerous diseases, mainly owing to their high target binding affinity and specificity. Unfortunately, antibodies are also limited in several respects, chief amongst those being the extremely high cost of manufacture. Therefore, non-antibody binding proteins have long been sought after as alternative therapies. New binding protein scaffolds are constantly being designed or discovered with some already approved for human use by the FDA. This review focuses on protein scaffolds that are either already being used in humans or are currently being evaluated in clinical trials. Although not all are expected to be approved, the significant benefits ensure that these molecules will continue to be investigated and developed as therapeutic alternatives to antibodies. Based on the location of the amino acids that mediate ligand binding, we place all the protein scaffolds under clinical development into two general categories: scaffolds with ligand-binding residues located in exposed flexible loops, and those with the binding residues located in protein secondary structures, such as α-helices. Scaffolds that fall under the first category include adnectins, anticalins, avimers, Fynomers, Kunitz domains, and knottins, while those belonging to the second category include affibodies, β-hairpin mimetics, and designed ankyrin repeat proteins (DARPins). Most of these scaffolds are thermostable and can be easily produced in microorganisms or completely synthesized chemically. In addition, many of these scaffolds derive from human proteins and thus possess very low immunogenic potential. Additional advantages and limitations of these protein scaffolds as therapeutics compared to antibodies will be discussed.

## Introduction

Antibodies have long been regarded as ‘magic bullets’ in human therapy due to their ability to bind targets with high affinity and specificity (Strebhardt and Ullrich, 2008). The first monoclonal antibody (mAb) entered human therapy in 1986. Since then, over 62 mAbs have been approved by the FDA as therapeutics and many new candidates are presently undergoing preclinical and clinical evaluations (Ecker et al., 2015; Reichert, 2017). However, antibodies are not without their limitations. For example, the large size of antibodies (~150 kDa) may impede their ability to penetrate into tumor tissue (Chauhan et al., 2011; Shah and Betts, 2013), and the planar binding interface makes it difficult to obtain antibodies that bind to grooves and catalytic sites of enzymes (Skerra, 2000). Despite its important role in prolonging the antibody half-life and recruiting immune effector cells, the antibody constant region—Fc—can sometimes give rise to adverse effects, such as antibody-dependent-enhancement (ADE) of infection by some viruses e.g., Dengue virus and Zika virus (Screaton et al., 2015; Dejnirattisai et al., 2016; Paul et al., 2016). In addition, most mAbs have to be produced in mammalian cells and often require post-translational modifications, such as specific glycosylation patterns. The astronomically high cost associated with therapeutic antibody production makes mAb-based therapeutics out-of-reach to most of the world’s population. Antibodies are also difficult to be manipulated for drug conjugation via the conventional conjugation and linker chemistries, as they are too big to be synthesized chemically and too complex to be produced in microorganisms. Finally, nearly all the current therapeutic mAbs are of murine origin, largely thanks to the hybridoma technology that enables the mouse B-cells to be immortalized and screened in clonal fashion for antigen-specific antibodies. Unfortunately, humans and mice are both mammals and many of our proteins/receptors share high homology. Homologous regions likely play important cellular functions and thus are preserved through evolution; hence, homologous regions are likely rich in therapeutic targets. Since self-antigens are not immunogenic for the host, it is difficult/impossible to obtain murine antibodies targeting homologous/identical regions from a human protein. This limitation also applies to other mammalian immunization hosts, although it can be alleviated to a large extent by generating antibodies in other non-mammalian species.

To overcome the limitations of antibodies, both non-antibody binding proteins (protein fragments) and antibody fragments (e.g., single-chain variable fragments (scFv), fragment antigen-binding (Fab) fragments, and single-domain antibody fragments (nanobodies)) have been designed and explored as scaffolds for therapeutic applications. Some of these have already been approved by the FDA for human use, and many are currently being evaluated pre-clinically or in clinical trials. Like antibodies, protein fragments can exert their therapeutic action through antagonizing receptors by inhibition of their ligand binding site(s), or binding to ligands to prevent their interaction with cognate receptor(s). These molecules can also function as antidotes and neutralize toxins or other harmful/infectious agents. The absence of an Fc may also prove to be beneficial in some cases to avoid the adverse effect associated with complement-dependent cytotoxicity (CDC) or antibody-dependent cellular cytotoxicity (ADCC). Unlike mAbs, protein fragments are generally very small in size (<20 kDa) and thus may be better able to penetrate tumors. In addition, many protein fragments exhibit high thermostability, allowing storage at room temperature for extended periods of time without significant loss of activity, and can be easily produced in microorganisms or be completely chemically synthesized, enabling facile functionalization with other drug or imaging agents. The low cost of production, combined with the ability of some molecules to resist protease degradation and/or chemical denaturation, makes it possible for some protein fragments to be used in oral applications. Further, almost all protein fragments are engineered completely *in vitro*, generating binders that are not biased in any way by exposure to other molecules *in vivo*. Finally, bispecific binders able to crosslink tumors cells with immune effector cells and/or different receptors can be easily generated from protein fragments. These bispecific binders are becoming a promising new class of protein therapeutics. Although it is possible to generate bispecific antibodies, their production is very complex, adding to the already high cost of mAb production. In contrast, multiple protein fragments can be easily linked, via genetic or chemical linkers, to form bispecific, or even multi-specific, binders.

**Figure 1:**
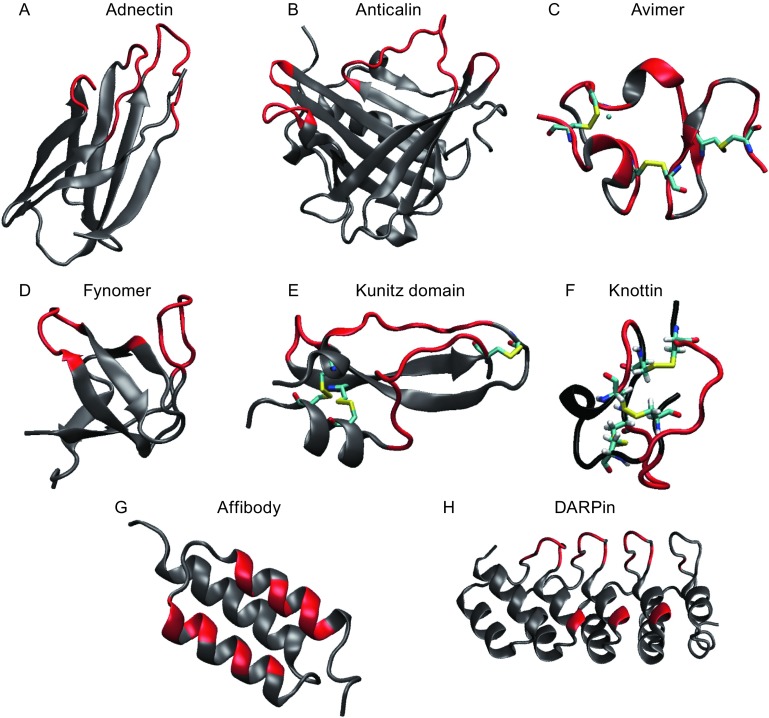
**Cartoon-structures of protein scaffolds.** The structures are displayed using visual molecular dynamics (VMD) (Humphrey et al., 1996). The loops that recognize the antigen are colored in red and the framework residues are indicated in gray. The disulfide bridges are indicated as sticks in element color and the calcium is represented as blue spheres. (A) adnectins (pdbcode: 1ttg), (B) Anticalin (pdbcode: 3BX7), (C) Avimer (pdbcode: 1ajj), (D) Fynomer (pdbcode: 1m27), (E) Kunitz domain (pdbcode: 1kth), (F) knottin (pdbcode: 2it7), (G) Affibody (pdbcode: 1q2n), (H) DARPin (pdbcode: 1mj0)

Many excellent reviews on mAb-alternative protein fragments have been published (Weidle et al., 2013; Jost and Pluckthun, 2014). In this paper, we focus on protein fragments that are either FDA-approved or are currently in human clinical development with special emphasis on those that have been engineered *in vitro*. These protein fragments are considered antibody-mimetic because, like antibodies, each protein fragment is composed of a constant region, which stabilizes the overall protein folding, and multiple variable regions that mediate its binding to a specific target. We place all the existing protein scaffolds into two general categories based on the location of the amino acids that mediate ligand binding: i) scaffolds with ligand-binding amino acids in exposed loops (Fig. 1A–F) and ii) those with these amino acids scattered in secondary structural motifs, such as α-helices (Fig. 1G–H). In the ensuing discussion, we will first discuss the various display platforms used for protein fragment engineering and their respective pros and cons, followed by examples of protein fragments that fall into both categories. Many protein fragments have been successfully engineered using multiple display platforms. The selection of different display platform was often based on the library size needed, the conditions for the selection, the ease of expression of the protein fragment, and intellectual property considerations.

## Display platforms for protein fragment engineering

Generally speaking, ligand-specific protein fragments have been engineered using a two-fold strategy: i) creation of a library of protein variants via targeted or random mutagenesis of the parent protein, and ii) selection of target ligand binders via a phenotypic selection such as phage display, yeast surface display or ribosome/mRNA display. The purpose of these display platforms is to establish a physical linkage between phenotype and genotype so that the sequences of the ligand-binding protein fragments can be deduced.

In phage display, the protein library is displayed as a fusion to either the phage gene 3 minor coat protein (P3), which is present at five copies on the surface of the filamentous M13 phage, or to the gene 8 major coat protein (P8) which is present at ~3,000 copies per phage particle (Sidhu et al., 2000). Proteins fused to P3 and P8 are typically displayed on the phage particle in a monovalent and polyvalent format, respectively. Fusion phage displaying a particular ligand-binding protein fragment can be isolated from other phage by their ability to bind to the ligand, a process called “panning”. The phage that are thus selected are subsequently amplified in *E*. *coli* and undergo additional rounds of panning, often at progressively increasing selection pressure (e.g., by using decreasing concentrations of ligand for later rounds of panning) to enrich the strongest ligand-binding protein fragments. Phage display has been a workhorse for protein engineering, thanks to the high stability of phage particles as well as the straightforward selection procedure. One limitation of phage display is its reliance on the transformation of *E*. *coli* with plasmids encoding the protein fragment library. Because of this requirement, the number of protein variants that can be subjected to the selection is limited by the transformation efficiency of the *E*. *coli*. Most phage displayed libraries have between 10^7^–10^9^ clones, although libraries of >10^10^ have been achieved. In addition, due to the prokaryotic nature of the *E*. *coli* translation and translocation machinery, not all protein fragments can be efficiently displayed on phage particles.

For the display of mammalian proteins that require endoplasmic reticulum-specific post-translational processing for efficient folding and activity, yeast has proved to be an attractive choice. In yeast surface display, the protein fragment is displayed on the surface of the yeast *Saccharomyces cerevisiae* as an N-terminal fusion to the Aga2p mating adhesion receptor which is anchored on the yeast cell wall via a pair of disulfide bridges to the Aga1 protein (Boder and Wittrup, 1997). Yeast-displayed protein libraries are typically subjected to selection using fluorescence-activated cell sorting (FACS) and/or affinity capture (Gai and Wittrup, 2007). However, as with phage display, the diversity of yeast display library is also limited by the efficiency with which the encoding DNA can be introduced into the microorganism. Most yeast-displayed libraries have 10^6^–10^8^ clones, although one study reported the selection of a library of >10^9^ clones (Feldhaus et al., 2003).

Unlike phage and yeast display, mRNA/ribosome display is a completely *in vitro* technology, obviating the need to transform cells in order to generate libraries and allowing the creation of libraries of >10^12^ different members. In ribosome display, a ternary complex composed of the translated proteins, the ribosome, and its encoding mRNA is used for selection (Dreier and Pluckthun, 2011). In mRNA display, the translated protein is covalently attached to the mRNA molecule via the adaptor puromycin molecule. The mRNA-protein adduct is subsequently purified from the ribosome and used for selection (Lipovsek and Pluckthun, 2004). After each round of selection, PCR amplification is employed to recover the selected mRNA to be used in the subsequent round. This integral PCR amplification step also provides a convenient opportunity for the introduction of additional diversity into the library. Both mRNA and ribosome display have been successfully used to identify low-pmol/L affinity binders, even after only a single round of selection (Schilling et al., 2014). The major limitation of this approach is that mRNA is an intrinsically unstable molecule. Since the stability of the mRNA must be maintained during the selection, mRNA/ribosome display necessitates that the selection be carried out under strict RNase-free conditions and at low temperature.

In the following sections we will discuss examples of ligand-binding protein fragments identified using phage display, yeast surface display, and ribosome/mRNA display.

## Category I: ligand-binding amino acids in exposed loops

Protein fragments with ligand-binding capacity in this category include adnectins, anticalins (affilins), avimers, Fynomers, Kunitz domains, and knottins.

### Adnectins

Adnectin is a 94-amino-acid thermostable (T_m_ > 80°C) binding protein fragment derived from the tenth domain of fibronectin type III (10Fn3), a human extracellular matrix protein (Lipovsek, 2011). The original function of 10Fn3 in fibronectin is to bind integrins. The molecule adopts a β-sandwich fold with seven strands connected by six loops, similar to an immunoglobulin domain but without any disulfide bonds. Three of the flexible loops on one side of the protein are surface-exposed and have proved to be a convenient interface for binding ligands of interest (Fig. 1A). Non-loop residues have also been found to expand the available binding footprint (Ramamurthy et al., 2012). Ligand-binding adnectin variants with binding affinities in the nanomolar to picomolar range have been selected via mRNA, phage, and yeast display (Hackel et al., 2008; Lipovsek, 2011).

The adnectin CT-322 was engineered via mRNA display to bind the vascular endothelial growth factor (VEGF) receptor with an affinity of 0.06 nmol/L (Getmanova et al., 2006). CT-322 was found to be effective at preventing tumor growth in a mouse model of pancreatic cancer (Dineen et al., 2008), and was PEGylated to improve its serum half-life *in vivo*. In a phase I clinical trial, PEGylated CT-322 was found to be well tolerated at doses up to 2 mg/kg and displayed a plasma half-life of up to 4 days (Tolcher et al., 2011). Unfortunately, in a subsequent phase II clinical trial, the CT-322 was found to be poorly effective, although the side effects were acceptable (Schiff et al., 2015).

The adnectin BMS-962476 was developed via mRNA display to bind cholesterol regulator proprotein convertase subtilisin/kexin-type 9 (PCSK9), an important therapeutic target for decreasing low-density lipoprotein (LDL) in cardiovascular disease. PEGylated BMS-962476 inhibited PCSK9 activity *in vitro* with an EC_50_ of 31 nmol/L and lowered the cholesterol levels in animal models (Mitchell et al., 2014). In a phase I clinical trial, BMS-962476 was found to be well-tolerated at doses up to 1 mg/kg and rapidly reduced free PCSK9 (>90%) and LDL levels (Stein et al., 2014).

BMS-986089 is an anti-myostatin adnectin that inhibits myostatin and GDF-11 second messenger signaling in cells. Myostatin is a negative regulator of skeletal muscle and BMS-986089 was developed as a potential treatment for skeletal muscle diseases such as Duchenne’s muscular dystrophy (DMD) (Madireddi et al., 2016). BMS-986089 is currently being evaluated in a phase II clinical trial (NCT02515669).

### Anticalins (Affilins)

Anticalin is a protein fragment derived from lipocalins, a class of secreted proteins that typically transport hydrophobic compounds (Skerra, 2008). The anticalin scaffold adopts a conserved β-barrel structure consisting of eight anti-parallel β-strands wound around a central axis and contains 160–180 amino acids (Fig. 1B). Anticalins are not glycosylated and do not possess any disulfide bonds. They are typically ~20 kDa in size, thermostable with melting temperatures >70°C and can be easily expressed in *E*. *coli* or yeast. The ligand-binding pocket is located near the surface of the protein and is composed of four extruding loops. A typical anticalin library used for identifying target ligand binders contains 16–24 randomized amino acids in each loop (Gebauer and Skerra, 2012). Ligand-specific anticalins have been engineered via phage display and bacterial surface display (Gebauer and Skerra, 2012).

PRS-050 is an anticalin engineered to bind vascular endothelial growth factor A (VEGF-A) via phage display and has been shown to block the interaction between VEGF-A and its cellular receptor with subnanomolar IC_50_ values (Gille et al., 2016). The *in vivo* half-life of PRS-050 was extended by site-directed PEGylation and the resulting modified anticalin effectively blocked VEGF-mediated growth of tumor xenografts in nude mice with a reduction in microvessel density (Gille et al., 2016). In a subsequent phase I clinical trial, PEGylated PRS-050 was found to be well-tolerated at a dose of at least 10 mg/kg and exhibited a half-life of up to 6 days following i.v. infusion (Mross et al., 2013a).

PRS-080 is a human hepcidin-25-binding anticalin. Hepcidin restricts iron availability in the blood, and PRS-080 was developed to mobilize irons trapped in iron storage cells in certain patients with anemia of chronic disease (ACD). PEGylated PRS-080 was found to be well-tolerated in a phase I clinical trial with a dose of at least 16 mg/kg and a plasma half-life of approximately 6 days following i.v. infusion (Pieris Pharmaceuticals, 2015).

### Avimers

Avimers are a class of binding protein fragments derived from the A-domain of various cell surface receptors such as the low density-related protein (LRP) and very low density lipoprotein receptor (VLDLR). Each A-domain has ~35 amino acids (~4 kDa) and adopts a uniform, stable structure stabilized by calcium binding and three pairs of disulfide bridges (Fig. 1C). The scaffold structure is maintained by 12 conserved amino acids, leaving all the remaining non-conserved residues amenable to randomization and ligand binding (Silverman et al., 2005). Avimers are highly thermostable and have been observed to be completely active after incubation at 50°C–80°C for two weeks (Weidle et al., 2013). Due to their small size, avimers often consist of multiple A-domains with each binding to a different site on the target. Avimers composed of up to eight A-domains have been generated and expressed soluble in the cytoplasm of *E*. *coli.*, despite the presence of three disulfide bonds per domain (Silverman et al., 2005).

The avimer C326 (AMG220), consisting of three A-domains, was engineered to bind IL-6 with picomolar affinity (Silverman et al., 2005). This molecule was evaluated in a phase I clinical trial (NCT00353756) for Crohn’s disease, but further development has since been halted.

### Fynomers

Fynomers are derived from amino acids 83–156 of the Src-homology 3 (SH3) domain of FYN tyrosine kinase (Cooke and Perlmutter, 1989). It is worth noting that FYN-SH3 domains are fully conserved between humans, mice, rats, and gibbons (Weidle et al., 2013), making these molecules non-immunogenic in humans. Each Fynomer is composed of a pair of anti-parallel beta sheets joined by two flexible loops which are the sites of ligand binding (Schlatter et al., 2012) (Fig. 1D). Fynomers are small (~7 kDa), thermostable (T_m_ ~70°C), and can be easily expressed in bacteria (Grabulovski et al., 2007).

The Fynomer 2C1 was engineered via phage display to bind the proinflammatory cytokine interleukin-17A (IL-17A) and was able to inhibit the activity of IL-17A *in vitro* with an IC_50_ of 2.2 nmol/L (Silacci et al., 2014). 2C1 was subsequently fused to the Fc domain of a human antibody to prolong its circulation half-life. Interestingly, the resulting dimeric 2C1-Fc (Fc is a dimer) exhibited >100-fold improved IC_50_ against IL-17A (21 pmol/L) compared to the parent 2C1 molecule and effectively inhibited IL-17A in a mouse model of acute inflammation (Silacci et al., 2014).

Inspired by the success of the 2C1-Fc fusion, the same group subsequently engineered FynomAb COVA322, a fusion molecule consisting of an IL-17A-binding Fynomer fused to the anti-TNF antibody adalimumab. FynomAb COVA322 was designed to simultaneously inhibit the activity of both TNF and IL-17A for treatment of rheumatoid arthritis (Silacci et al., 2016). Bispecific FynomAb COVA322 inhibited IL-17A and TNF with *in vitro* IC_50_ values of 121 pmol/L and 169 pmol/L, respectively and was effective *in vivo* (Silacci et al., 2016). COVA322 is currently being evaluated in a phase I/II clinical trial (NCT02243787).

### Kunitz domains

Kunitz domains are ~60-amino-acid peptides (~7 kDa) derived from the active motif of Kunitz-type protease inhibitors such as aprotinin (bovine pancreatic trypsin inhibitor), Alzheimer’s amyloid precursor protein, and tissue factor pathway inhibitor (Bode and Huber, 1992). The hydrophobic core of the Kunitz domain is composed of a twisted two-stranded antiparallel β-sheet and two α-helices stabilized by three pairs of disulfide bonds (Fig. 1E). Residues in the three loops can be substituted without destabilizing the structural framework (Hosse et al., 2006).

Ecallantide (DX-88), a Kunitz domain-derived inhibitor of kallikrein (a subgroup of serine proteases), was approved by the FDA in 2012 for treatment of hereditary angioedema (HAE), a rare, autosomal dominantly inherited blood disorder that manifests as an episodic swelling of the body (Schneider et al., 2007; Cicardi et al., 2010; Levy et al., 2010). Most HAE is caused by the malfunction of the plasma C1 kallikrein inhibitor protein, and can thus be treated with a substitute kallikrein inhibitor—Ecallantide—during acute HAE attacks (Nussberger et al., 1998). DX-88 was derived from the Kunitz domain of lipoprotein-associated coagulation inhibitor (LACI) and was engineered to bind kallikrein with low picomolar affinity via phage display (Williams and Baird, 2003).

The Kunitz domain peptide Depelstat (DX890) is a potent and selective inhibitor of human neutrophil elastase (*K*_d_ = 1 pmol/L) (Roberts et al., 1992). Inflammation mediated by neutrophil elastase contributes to lung damage in cystic fibrosis. DX890 was shown to reduce neutrophil trans-epithelia migration and inflammation *ex vivo* (Dunlevy et al., 2012) and has been evaluated in a phase II clinical trial for the treatment of cystic fibrosis (NCT00455767).

### Knottins (Cysteine knot miniproteins)

A knottin is an extremely stable 30-amino-acid protein fold (<4 kDa) composed of three anti-parallel β-strands connected by loops of variable length and multiple disulfide bonds (Fig. 1F). A unique characteristic of knottins is the so-called cysteine knot where a disulfide bond crosses the macrocycle formed by the other disulfides. A subclass of knottin is the cyclotides in which the N- and C-terminus of the protein is joined post-translationally to form a circular molecule (Craik et al., 2010). The cysteine knot framework provides knottins with extraordinary thermic, proteolytic, and chemical stability (Colgrave and Craik, 2004; Werle et al., 2006; Kintzing and Cochran, 2016b). The melting temperature for most knottins is >80°C. The high proteolytic stability confers knottins with the ability to survive the harsh conditions of the gut, thus making these molecules viable candidates for oral administration (Wong et al., 2012; Thell et al., 2016). The small size and high stability of knottins allows the molecules to be conveniently produced via chemical synthesis and high-yield expression in microbial hosts (Schmoldt et al., 2005; Avrutina, 2016). Naturally occurring knottins are found in a wide range of species including plants, animals and fungi, and mediate a wide range of functions including protease inhibition, ion channel blockade, and antimicrobial activity (Zhu et al., 2003; Gracy et al., 2008; Aboye et al., 2015; Tam et al., 2015). The surface-exposed loops of knottin have been extensively engineered for ligand binding (Kintzing and Cochran, 2016a).

Ziconotide (marketed as Prialt) is a naturally derived knottin peptide found in the venom of the fish-eating marine cone snail, *Conus magnus*. This peptide is a component of the venom used by the animals to immobilize its prey. Ziconotide was approved by the FDA in 2004 for the treatment of severe chronic pain (Smith and Deer, 2009). Ziconotide binds and antagonizes the N-type voltage-sensitive calcium channels (NVSCCs) abundant in nerves involved in pain signaling with low picomolar affinity (Kristipati et al., 1994). In a rat model of neuropathic pain, Ziconotide was found to be more effective than morphine (Wang et al., 2000; Smith and Deer, 2009). Ziconotide is approved for intrathecal administration to patients who experience severe chronic pain and who are refractory to other treatments (Rauck et al., 2006; Wallace et al., 2006).

Linaclotide (marked as Linzess) is another naturally-derived knottin that was approved by the FDA in 2012 to treat Irritable Bowel Syndrome with Constipation (IBS-C) and Chronic Idiopathic Constipation (CIC) (Layer and Stanghellini, 2014). Linaclotide is a high-affinity agonist of guanylate cyclase-C (GC-C) (Chey et al., 2012). Activation of GC-C in the intestinal lumen initiates a signal transduction cascade that results in the secretion of chloride and bicarbonate. In rodent models, oral administration of linaclotide resulted in increased gastrointestinal transit and reduced visceral pain (Bryant et al., 2010; Eutamene et al., 2010).

## Category II: ligand-binding amino acids in secondary structure

Protein fragments with ligand-binding ability in this category that are currently under clinical development include affibodies, β-hairpin mimetics, and DARPins.

### Affibodies

Affibodies are protein fragments derived from the Z-domain of the Ig-binding region of *Staphylococcus aureus* protein A (Nygren, 2008) which adopt a three-helix bundle motif and contain no cysteines (Fig. 1G) (Nord et al., 1997). These molecules possess high thermal and proteolytic stability and can be easily expressed in *E*. *coli.* The ligand-binding surface is composed of 13 solvent-accessible residues scattered among two of the helices. The small size (58 amino acids, 7 kDa) of affibodies allow them to be produced by chemical synthesis. Affibodies exhibit rapid extravasation and rapid tumor penetration and unbound affibodies are quickly cleared from healthy organs and tissues, making them promising reagents for radionuclide imaging (Ahlgren and Tolmachev, 2010).

The affibody ABY-025 was engineered via phage display and affinity maturation to bind HER2 with low picomolar affinity (Nord et al., 1996). The scaffold region of ABY-025 was subsequently optimized to provide improved thermal and chemical stability and hydrophilicity (Feldwisch et al., 2010). In a phase I/II clinical trial, ^68^Ga-gallium labelled ABY-025 ([^68^Ga]ABY-025) was able to accurately quantify HER2-receptor status in metastatic breast cancer via positron emission tomography (PEG) imaging (Sandstrom et al., 2016; Sorensen et al., 2016).

### β-Hairpin mimetics

β-Hairpin mimetics, as the name suggests, comprise a single β-hairpin motif designed to reproduce the conformational and electronic properties of functional native protein epitopes (so-called protein epitope mimetics (PEM)) (Fasan et al., 2004). PEMs are often cyclic, very small in size (1–2 kDa) and contain multiple disulfide bonds to stabilize the protein fold.

POL5551 is a β-hairpin mimetic selected to antagonize CXCR4 for the mobilization of hematopoietic stem cells (Karpova et al., 2013). High CXCR4 expression levels also correlate with tumor metastasis, and POL5551 was later shown to reduce the metastasis of triple-negative breast cancer in mice when combined with eribulin, a chemotherapeutic microtubule inhibitor (Xiang et al., 2015). POL6326, an analogue of POL5551, is currently being evaluated for breast cancer treatment in a phase I clinical trial in combination with eribulin (NCT01837095).

### DARPins

Designed ankyrin repeat proteins (DARPins) are artificial protein scaffolds based on ankyrin repeat (AR) proteins which mediate diverse protein-protein interactions in virtually all species (Bork, 1993). Most natural AR proteins contain 4–6 AR domains stacked onto each other (Walker et al., 2000). DARPins contain 2–3 internal ARs sandwiched between the N- and C-terminal capping repeats. Each internal AR module consists of 27 defined framework residues and 6 potential protein-binding residues that form a β-turn followed by two antiparallel helices and a loop connecting to the β-turn of the next repeat (Binz et al., 2003) (Fig. 1H). DARPins are small in size (14–18 kDa, depending on the number of internal ARs), thermostable (T_m_ up to 90°C), resistant to proteases and chemical denaturants, and can be expressed to very high levels in *E*. *coli* (up to 200 mg per liter of shake flask culture) (Pluckthun, 2015). Last but not least, DARPins have a relatively large binding interface and have been engineered, mostly via phage display and ribosome display, to bind a wide range of targets with pmol/L–nmol/L affinities (Pluckthun, 2015).

The DARPin MP0112 was engineered to bind VEGF-A with a *K*_d_ of 1–4 pmol/L (Souied et al., 2014b). MP0112 was tested in a series of clinical trials for treating age-related macular degeneration (AMD) and diabetic macular edema (DME), both of which are eye conditions that can cause significant vision impairment. Although the pathogenesis of these diseases is not completely understood, VEGF antagonists have been shown to retard the disease progression (Ferrara et al., 2006). MP0112 demonstrated encouraging results in phase I/II studies. In the DME trial, MP0112 was well-tolerated in patients and exhibited an ocular half-life of more than 13 days (Campochiaro et al., 2013). A single intraocular injection of 0.4 mg MP0112 neutralized VEGF in aqueous humor for 8–12 weeks (Campochiaro et al., 2013). Inflammation was reported for some patients, ostensibly due to impurities present in the protein preparation purified from the *E*. *coli* culture (Campochiaro et al., 2013). Similarly, in the AMD trial, MP0112 was effective for up to 8 weeks following a single intraocular dosage with inflammation reported in some patients (Souied et al., 2014a). Subsequently, the protein purification process was improved, and MP0112 was reformulated to contain a PEG molecule and renamed as Abicipar Pegol. Phase I/II trials of Abicipar Pegol showed lower incidence of inflammation when compared to the trials using MP0112. Abicipar Pegol is currently being evaluated in two phase III trials for AMD (NCT02462486, NCT02462928).

MP0250 is a multi-DARPin trispecific molecule able to neutralize the activities of VEGF and hepatocyte growth factor (HGF) simultaneously. The molecule is also able to bind human serum albumin (HSA), conferring it with an increased serum half-life and potentially enhanced tumor penetration. In a phase I clinical trial, MP0250 was found to be well-tolerated after i.v. infusion at a dose of at least 8 mg/kg and a median half-life of ~12 days (Molecular Partners, 2015; Rodon et al., 2015).

MP0274 is another multimer composed of two DARPins that bind to distinct epitopes on the human epidermal growth factor receptor 2 (HER2) and inhibits downstream HER2- and HER3-mediated signaling. MP0274 demonstrated good efficacy in preclinical models (Reichert et al., 2014) and a phase I trial is planned for 1Q 2017 (Table 1).

**Table 1:** Protein fragments currently approved by the FDA or in clinical trials

Scaffold	Name	Affinity	Molecular target	Disease targeted	Company	Clinical trials
Adnectin	CT-322	0.06 nmol/L	VEGF receptor	Pancreatic cancer	Bristol-Meyers Squibb	Phase I, Phase II (Tolcher et al., 2011; Schiff et al., 2015)
	BMS-962476	0.85 nmol/L	PCSK9	Hypercholestoremia	Bristol-Meyers Squibb	Phase I (Stein et al., 2014)
	BMS-986089	0.17 nmol/L	Myostatin	Duchenne muscular dystrophy	Bristol-Meyers Squibb	Phase I (NCT02145234) Phase II (NCT02515669)
Affibody	ABY-025	76 pmol/L	HER2	Tumor imaging	Affibody	Phase I (NCT02095210) Phase I, II (NCT01858116, NCT01216033)
Anticalin	PRS-050	<1 nmol/L	VEGF	Tumor suppression	Pieris	Phase I (Mross et al., 2013b)
	PRS-080	50 pmol/L	Hepcidin	Anemia	Pieris	Phase I (Moebius et al., 2015) Phase I/II (NCT02754167)
Avimer	C326 (AMG220)	<0.2 nmol/L	IL-6	Crohn’s disease	Amgen	Phase I (NCT00353756)
DARPin	MP0112	2 pmol/L	VEGF-A	AMD, DME	Molecular Partners, Allergan	Phase I/II (Campochiaro et al., 2013; Souied et al., 2014a).
	Abicipar	2 pmol/L	VEGF-A	AMD	Molecular Partners, Allergan	Phase III (NCT02462486, NCT02462928)
	MP0250	<1 nmol/L	VEGF, HGF	Tumor suppressor	Molecular Partners, Allergan	Phase I (Rodon et al., 2015)
	MP0274		HER2	Tumor suppressor	Molecular Partners, Allergan	Phase 1 (Reichert et al., 2014)
Fynomer	COVA 322	0.9 nmol/L	Chymase	Plaque psoriasis	Covagen	Phase I/II (NCT02243787)
Knottin	Ziconotide (Prialt)	1 pmol/L	N-type calcium channels	Neuropathic pain	Jazz Pharmaceuticals	FDA approval in 2004
	Linaclotide (Linzess)	1 nmol/L	Guanylate cyclase C receptor	Irritable bowel disease	Ironwood Pharmaceuticals	FDA approval in 2012
Kunitz domain	DX-88 (Ecallantide)	44 pmol/L	Plasm kallikrein	Hereditary angioedema	Dyax	FDA approval in 2012
	DX-890 (Depelstat)	1 pmol/L	Neutrophil elastase	Pulmonary fibrosis	Dyax	Phase II (NCT00455767)
β-hairpin mimetics	POL6326	2 nmol/L	CXCR4	Tumor suppressor	Polyphor	Phase I (NCT01837095)

## Outlook

The existence of several non-antibody protein fragments in clinical studies certainly points to the promise of these molecules in human therapy. Nonetheless, there are challenges associated with using non-antibody binding proteins as therapeutics. One is immunogenicity, as all non-host proteins are potentially immunogenic and carry the risk of being rejected by the host. However, even fully human antibodies can be immunogenic in human patients, as found for adalimumab (Humira) (Bender et al., 2007), and each individual case needs to be evaluated independently. Not surprisingly, most of the protein fragments currently under clinical development are either derived from human proteins (e.g., adnectins, anticalins, avimers, Fynomers, and Kunitz domains) or possess a low immunogenic potential (e.g., DARPins (Pluckthun, 2015) and knottins (Moore and Cochran, 2012)) likely due to inefficient peptide-MHC presentation to the immune system (Maillere et al., 1995). Molecules that are potentially immunogenic (e.g., affibodies) are being largely developed for short-term imaging rather than for therapeutic applications.

Another concern with antibody mimetics is the short *in vivo* half-life. Most protein fragment therapeutics fall below the 70 kDa threshold for glomerular filtration (Caliceti and Veronese, 2003). Several strategies have been developed to extend the protein fragment serum half-life, such as PEGylation (Veronese and Pasut, 2005; Bailon and Won, 2009) and association or covalent conjugation with serum albumin (Smith et al., 2001; Dennis et al., 2002; Nguyen et al., 2006; Holt et al., 2008; Elsadek and Kratz, 2012) or an antibody Fc domain (Kontermann, 2011; Angelini et al., 2012). However, none of these strategies are able to extend the half-life of protein fragments to that of native serum proteins such as antibodies and serum albumin, both of which have a serum half-life of ~21 days (Chaudhury et al., 2006). While a short half-life is not necessarily a disadvantage for the treatment of acute conditions, it represents a challenge for chronic or recurring illnesses.

Finally, like antibodies, most protein fragments cannot be administered orally. The acidic environment of the stomach, in addition to the activity of proteases in the stomach and small intestine, make it difficult for most protein fragments to make it through the digestive tract intact. Exceptions are molecules that possess extremely a high proteolytic and chemical stability, such as knottins (Kolmar, 2009) and potentially DARPins.

Despite the limitations of non-antibody protein binding domains, the challenges associated with therapeutic mAb development, such as issues with host selection for generation of mAbs, humanization, high cost of manufacture, potentially poor tissue penetration, and ADE of viral infection, etc., ensures that therapeutic protein substitutes for mAbs will continue to be sought after and developed for the foreseeable future. In addition, mAb therapeutics has a high manufacturing cost and is currently mostly targeted to patients living in developed countries (Kelley, 2009; Sparrow et al., 2017). The manufacturing cost of non-antibody protein fragments can be significantly lower, largely stemming from their production in microbial hosts. The development of non-antibody protein therapeutics may therefore be more economically feasible for patients, especially those suffering from viral or bacterial infection, in developing countries.
